# Analysis of Population Differences in Digital Conversations About Cancer Clinical Trials: Advanced Data Mining and Extraction Study

**DOI:** 10.2196/25621

**Published:** 2021-09-23

**Authors:** Edith A Perez, Elizabeth M Jaffee, John Whyte, Cheryl A Boyce, John D Carpten, Guillermina Lozano, Raymond M Williams, Karen M Winkfield, David Bernstein, Sung Poblete

**Affiliations:** 1 Division of Hematology and Oncology Mayo Clinic Jacksonville, FL United States; 2 Sidney Kimmel Comprehensive Cancer Center Johns Hopkins University Baltimore, MD United States; 3 WebMD New York, NY United States; 4 Ohio Commission on Minority Health Columbus, OH United States; 5 Institute of Translational Genomics Keck School of Medicine University of Southern California Los Angeles, CA United States; 6 Department of Genetics The University of Texas MD Anderson Cancer Center Houston, TX United States; 7 DLA Piper Philadelphia, PA United States; 8 Meharry-Vanderbilt Alliance Vanderbilt University Medical Center Nashville, TN United States; 9 Stand Up To Cancer Los Angeles, CA United States

**Keywords:** cancer, clinical trials, data mining, text extraction, social media, race and ethnicity, health communication, health care disparities, natural language processing

## Abstract

**Background:**

Racial and ethnic diversity in clinical trials for cancer treatment is essential for the development of treatments that are effective for all patients and for identifying potential differences in toxicity between different demographics. Mining of social media discussions about clinical trials has been used previously to identify patient barriers to enrollment in clinical trials; however, a comprehensive breakdown of sentiments and barriers by various racial and ethnic groups is lacking.

**Objective:**

The aim of this study is to use an innovative methodology to analyze web-based conversations about cancer clinical trials and to identify and compare conversation topics, barriers, and sentiments between different racial and ethnic populations.

**Methods:**

We analyzed 372,283 web-based conversations about cancer clinical trials, of which 179,339 (48.17%) of the discussions had identifiable race information about the individual posting the conversations. Using sophisticated machine learning software and analyses, we were able to identify key sentiments and feelings, topics of interest, and barriers to clinical trials across racial groups. The stage of treatment could also be identified in many of the discussions, allowing for a unique insight into how the sentiments and challenges of patients change throughout the treatment process for each racial group.

**Results:**

We observed that only 4.01% (372,283/9,284,284) of cancer-related discussions referenced clinical trials. Within these discussions, topics of interest and identified clinical trial barriers discussed by all racial and ethnic groups throughout the treatment process included health care professional interactions, cost of care, fear, anxiety and lack of awareness, risks, treatment experiences, and the clinical trial enrollment process. Health care professional interactions, cost of care, and enrollment processes were notably discussed more frequently in minority populations. Other minor variations in the frequency of discussion topics between ethnic and racial groups throughout the treatment process were identified.

**Conclusions:**

This study demonstrates the power of digital search technology in health care research. The results are also valuable for identifying the ideal content and timing for the delivery of clinical trial information and resources for different racial and ethnic groups.

## Introduction

### Background

The internet age has opened up a wealth of web-based health-related information for patient access. Most recently, the increased use of social media has allowed patients to use blogs, web-based forums, and support groups for education, support, and connection with other patients undergoing similar health care experiences. New data mining technologies have allowed us to use this wealth of information to gain valuable and timely insights into areas such as adverse drug effects [1-3] and the spread of infectious diseases [4,5]. Advancements in natural language processing and machine learning algorithms now allow for the examination of patient ideas, sentiments, and feelings about a range of topics [6,7]. This is known as sentiment analysis and is being increasingly used in the health care domain to gain unfiltered insight into patient satisfaction and efficacy of care from web-based patient input [8,9]. This information is useful for health care providers to optimize their services and improve patient care.

The Pew Research Center, which has tracked the demographics of internet users since 2005, has found comparable social media use between different racial and ethnic groups. As of June 2019, 73% of White, 69% of Black, and 70% of Hispanic people regularly used social media [10]. However, although their use time is similar, the details of the health care sites visited and information sought and shared by different races or ethnic groups have not been thoroughly examined. The race or ethnicity of the poster is not always evident in social media posts, but when it is provided, researchers gain the opportunity to sort these discussions by demographics and gain valuable insights into health care topics and barriers relevant to each group.

### Study Goals

In this study, we focus our analysis on the differing trends in discussions of cancer clinical trials between different racial or ethnic groups of social media users. Currently, there is a disconnect between the large number of available clinical trials testing potentially active new drugs and the relatively small number of patients with cancer willing to enroll in these clinical trials. The national average is well under 10% of all patients with cancer enrolled in clinical trials. Furthermore, recent studies have shown that enrollment into cancer clinical trials in the United States between 2010 and 2016 underrepresented some racial and ethnic minority groups [11]. This finding is alarming because as many as 20% of new drugs being tested in clinical trials can have different pharmacokinetics, pharmacodynamics, and safety profiles among different racial and ethnic groups, which can lead to disparities in treatment response, morbidity, and mortality, leading to trial results that are not indicative of the patient population [12,13]. These realities necessitate that clinical trials evaluating new therapies include a diverse population with the necessary numbers of ethnic minority participants to detect differences in these outcomes and provide equitable health care for all.

Social media is increasingly being recognized for its potential to connect patients with clinical trial information and education, aid in recruitment, and identify patient concerns and barriers to enrollment [14]. In a recent study, Peng et al [15] mined discussions from web-based cancer forums to identify clinical trial sentiments, priority areas of discussion, barriers, and opportunities for patient outreach. Many of these social media studies, however, analyze conversations from limited, targeted cancer-related sites and lack demographic information on the individuals posting the conversations. In this study, we used a powerful research method to search for patient discussions about clinical trials across the internet. This is an innovative discovery-based approach in which topics emerge from conversations instead of preimposing topics to mine. The large number of discussions we found allowed us to extract a substantial subset of conversations with identifiable race or ethnicity of the poster and to examine the similarities and differences in their thoughts and ideas on cancer clinical trials by sentiment analysis. Data were further categorized by treatment stage, which provides additional insight into the relationship of each group with the clinical trial process. This is a demonstration of the usefulness of this technology in health care research, and the results may be valuable for tailoring clinical trial education, enrollment, and delivery to various racial and ethnic groups.

## Methods

### CulturIntel Search Methodology

CultureIntel, a data science affiliate of CIEN+, has developed a novel methodology that mines unstructured qualitative data. Advanced search techniques such as web spiders, crawlers, and site scraping are able to *listen* to web-based conversations about cancer clinical trials and extract topical information and tagged data into a database. By not preselecting sites for analysis, we are able to look at the full universe of conversations that are available to gain unbiased and spontaneous insights into our topic of interest. This technology has been used previously to understand barriers to the treatment of women of color living with breast cancer [16] and to examine suicide-related digital conversations among teenagers and adults with epilepsy [17]. The CulturIntel methodology is 100% compliant with the General Data Protection Regulation requirements. All the conversations collected were open-source, public conversations. The data content was anonymous and not stored after the analysis.

### Data Collection

This analysis was conducted on digital conversations in the English language from IP addresses in the United States for a 12-month period ending on June 21, 2018.

### Sites and Users

**Figure 1:**
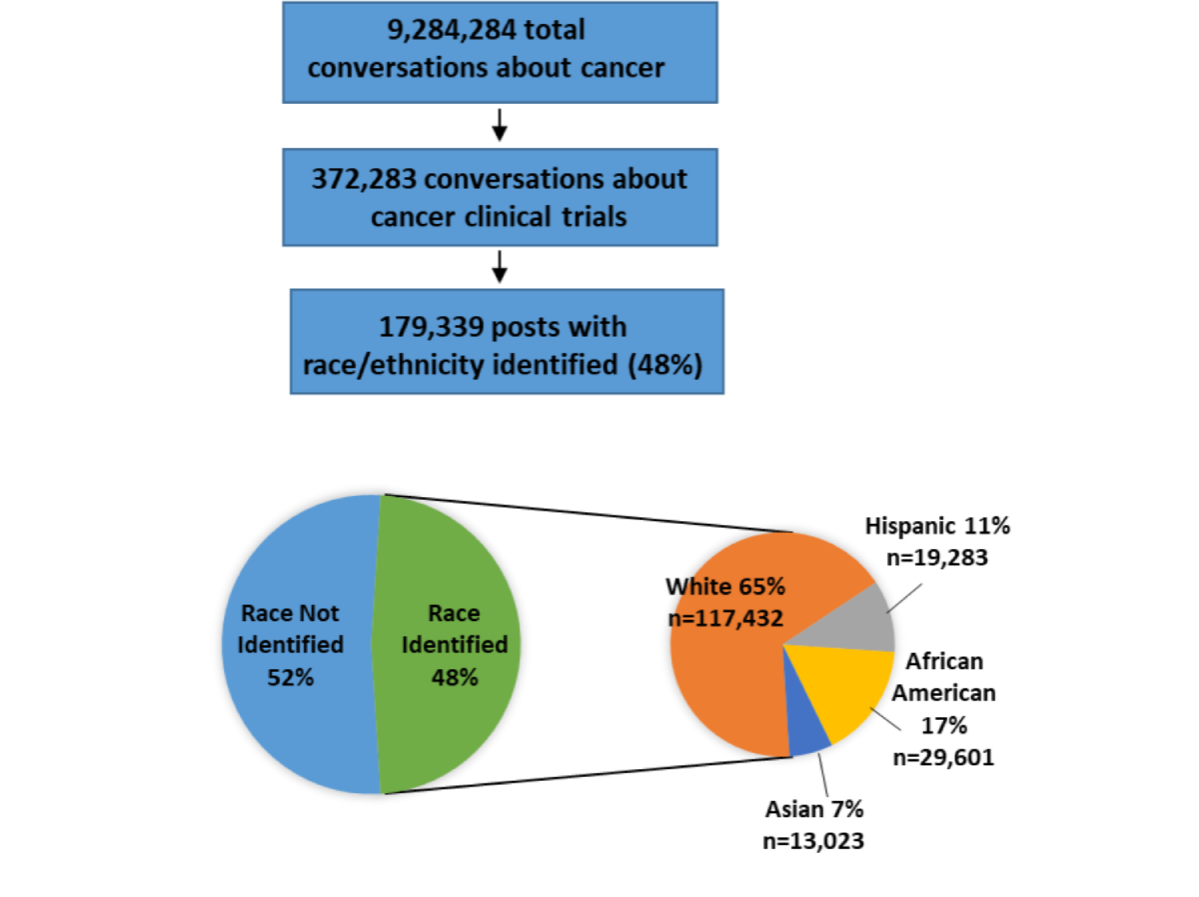
Description of online discussions included in the data and demographic categorization of analyzed posts.

Conversations were primarily found on message boards and other topical sites that numbered in the tens of thousands. Users could have more than one post included if they were part of a unique post. Multiple user posts within a conversation and shared or linked comments were counted once, whereas users’ posts across different discussions or sites were counted separately. A total of 9,284,284 conversations about cancer were identified, of which 372,283 (4.01%) were related to cancer clinical trials. Of these 372,283 discussions, 179,339 (48.17%) had identifiable racial or ethnic information. The racial or ethnic distributions are shown in Figure 1. When the race or ethnicity of a user could not be identified, it was still included in the *overall* results.

### Content Analysis

Natural language processing, text analytics, artificial intelligence, and social data mining were used to extract information from the collected conversations, including (1) the relationship of the posting individual to the patient, (2) the type of post or question, (3) the sentiment or feelings of the poster, (4) stage of cancer treatment from post semantics, (4) topics of discussion, and (5) perceived barriers. Topics, sentiments, and stages were not preselected but rather emerged from the data. The study protocol, algorithm parameters, and checks to ensure the relevance of the data extracted were all performed by an in-person study team. Sentiment analysis was also human supervised to ensure the accuracy of the attribution of the sentiment to a conversation.

## Results

### Content Demographics

We conducted a comprehensive web-based search for conversations about cancer clinical trials over a 12-month period ending on June 21, 2018. A total of 9,284,284 conversations about cancer were detected, with 372,283 referencing clinical trials. Of the 372,283 clinical trial conversations, 179,339 (48.17%) were posted by individuals with identifiable race or ethnicity. Of these 179,339 conversations, 117,432 (65.48%) were posted by individuals categorized as White, 19,283 (10.75%) by Hispanics, 29,601 (16.51%) by African Americans, and 13,023 (7.26%) by Asians (Figure 1). These conversations were found on disease-related topical sites (134,483/372,283, 36.12%), social networks (52,329/372,283, 14.06%), message boards (152,184/372,283, 40.88%), and blogs (33,092/372,283, 8.89%).

**Figure 2:**
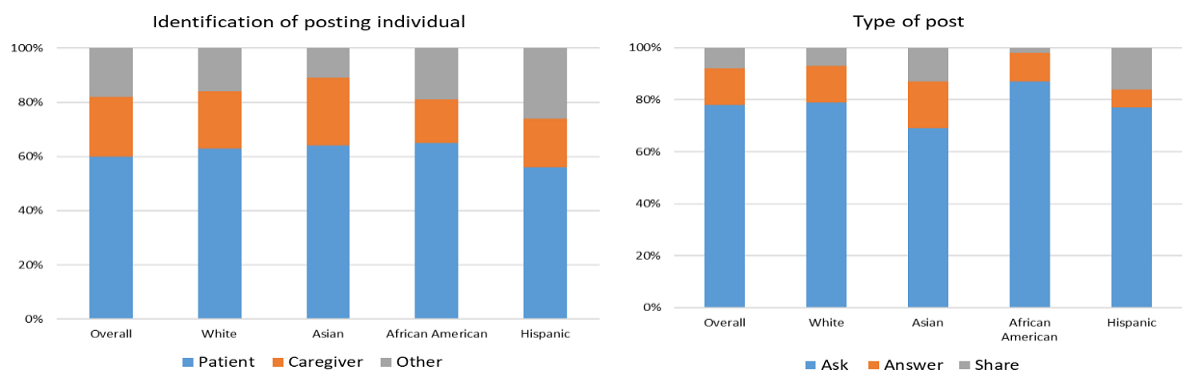
Analysis of posting individuals and type of post.

Overall, 60% (223,384/372,283) of the posts were from the patients themselves, 22.05% (82,103/372,283) were from a caregiver, and 18.22% (67,283/372,283) were from another individual and classified as *other*. These distributions were similar across ethnic groups, only varying slightly in the *other* category, with Hispanics having more and Asians having fewer posts by individuals in this category (Figure 2). The vast majority of the posts analyzed for “type of post” (214,009/276,069, 77.52%) were questions seeking information (ie, “...I know some of my forum will have the valuable experiences that may assist as to what direction I take in terms of considering trials?”), whereas 14.43% (39,829/276,069) were answering questions (ie, “Treatments were painless and quick and the staff was lovely.”) and 8.01% (22,231/276,069) were sharing information or support (ie, “There are lots of great people on this site who are both living with cancer and have a loved one with cancer. They have been instrumental in helping me get through the experience of dealing with the trials process”). The posts by African Americans were more likely to be questions than posts from other groups, while posts by Hispanics were twice as likely to share information and support (Figure 2).

### Sentiment Analysis

**Figure 3:**
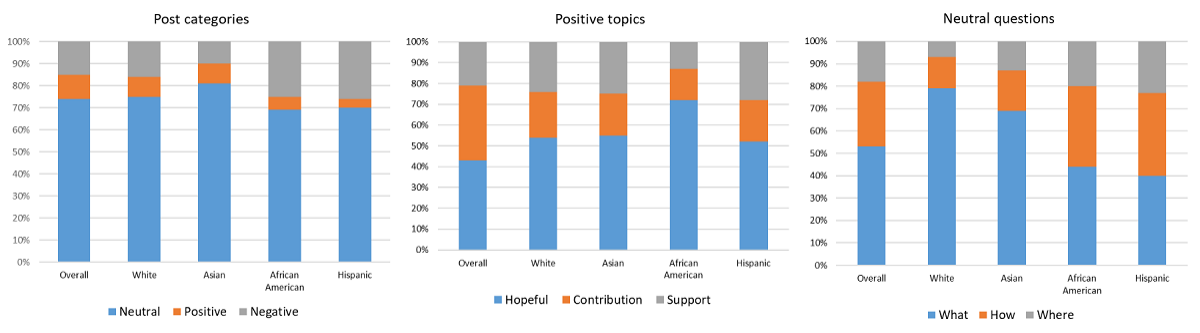
Categorization of post sentiments and details of positive posts and neutral questions.

Sentiment analysis, also called emotion artificial intelligence or opinion mining, is a method used to analyze natural language processing, computational linguistics, texts, and biometrics. This method is often used to analyze information collected from web-based social media. We applied sentiment analysis to study the data we collected and categorize attitudes, opinions, and reactions to cancer clinical trial–related information by race and ethnicity. With our analysis methods, we found that 74.07% (275,659/372,156) of the posts with identifiable sentiment were neutral in nature (ie, “What are the possible risks, side effects, and benefits of the study treatment compared to my current treatment?”), 15.12% (56,274/372,156) were negative (ie, “...its so much regret with which I wake up everyday and feel so sad I trusted these doctors”), and 10.81% (40,223/372,156) were positive (ie “I’m very excited to participate in a clinical trial”). The results were relatively similar across the different groups, although African Americans and Hispanics had slightly more negative posts than the overall population, whereas Asians posted a higher percentage of neutral posts (Figure 3). The negative topics includes lack of awareness (145,090/372,283, 38.97%), fear and anxiety (115,563/372,283, 31.04%), and concerns about health care professionals (HCPs; 41,235/372,283, 11.08%), costs (40,092/372,283, 10.77%), and logistics (31,674/372,283, 8.51%). Positive posts were categorized as hopeful, contributory, or grateful for the support as illustrated in Figure 3. Posts by African Americans were the most hopeful, whereas Hispanics expressed the most gratefulness for support (Figure 3).

Most posts across all groups were neutral in nature. These posts were primarily questions and are categorized in Figure 3 as *what*, *how*, and *where* questions. White individuals and Asians asked more *what* questions, that is, seeking information about clinical trials, whereas African Americans and Hispanics asked more logistical questions about the *where* and *how* information pertaining to clinical trial access (Figure 3).

**Figure 4:**
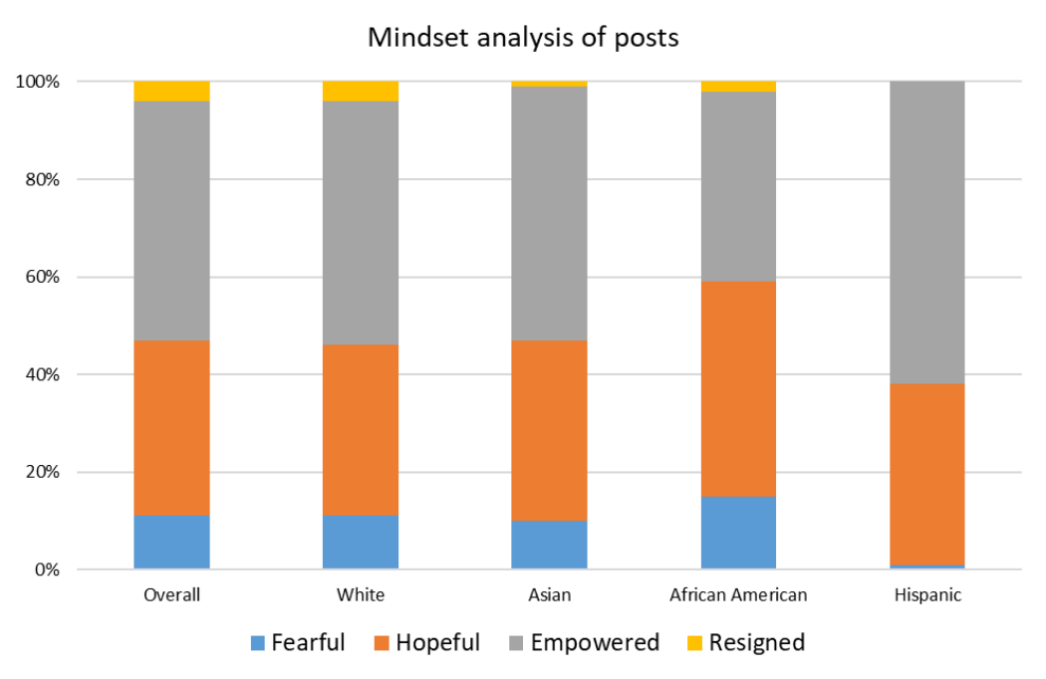
Mindset analysis of posts by race or ethnicity.

Mindset sentiments were also analyzed and are summarized in Figure 4. Posts were designated as *fearful* (41,371/372,283, 11.11%; ie, “I am trying to find ways to cope with this news. 3 days ago I was diagnosed with stage 4 lung cancer. I am completely devastated.”), *hopeful* (134,901/372,283, 36.24%); (ie, “I have faith in god and trust in my doctors that I will be cured.”), *empowered* (182,481/372,283, 49.02%); (ie, “I will do anything and everything it will take to beat this!”), or *resigned* (15,381/372,283, 4.13%); (ie, “I have always heard lung cancer is a death sentence and survival is nil. I am wondering where things go from here.”). The distribution of these sentiments was very similar across racial groups, although posts by Hispanics were more often categorized as *empowered* (12,030/19,115, 62.93% vs 182,481/372,283, 49.02%). Very few posts were categorized as resigned or fearful (Figure 4).

### Treatment Stages

CultureIntel used human-assisted text mining to categorize 4 stages of cancer treatment the referenced patient was currently undergoing. The *coping* stage was defined as coming to grips with disease realities and impacts (ie, “...polyps can appear one year and also take some time (years) to become cancerous.”); the *treating* stage was defined as enduring the treatment process (ie, “They want to do a Selective Internal Radiation Therapy along with continued Chemo. Has anyone had this done before?”); the *monitoring* stage was defined as ongoing evaluation of treatment success or efficacy and disease status (ie, “...and the oncologist is now suggesting Lonsurf. My understanding of Lonsurf is that this is a matter of buying her a few more months...”); and the *adjusting* stage was defined as changes or stabilization of disease state and/or treatment plan (ie, “I was diagnosed with breast cancer 3 weeks ago...now I am told I have a spot on one of my lungs...I am so scared”). The distribution of posts from each racial or ethnic group during the 4 treatment stages is shown in Table 1. Of particular note, we found that Hispanics do not often share posts in the sites analyzed at the coping stage. The reasons for this are not known, but it is possible that this population prefers to seek and share information on the web when they are further along in the treatment process (Table 1).

**Table 1:** Discussions at stages of treatment.

Race or ethnicity	Coping, n (%)	Treating, n (%)	Monitoring, n (%)	Adjusting, n (%)
Overall	17,443 (19.12)	97,332 (26.05)	78,487 (21.01)	126,343 (33.82)
White individuals	20,182 (16.81)	34,723 (28.93)	25,311 (21.09)	39,809 (33.17)
Asians	1937 (12.41)	5215 (33.4)	3095 (19.82)	5365 (34.36)
African Americans	3009 (10.03)	13,876 (46.28)	6674 (22.26)	6422 (21.42)
Hispanics	0 (0)	8456 (41.49)	7812 (38.33)	4112 (20.18)

### Discussion Topics

**Figure 5:**
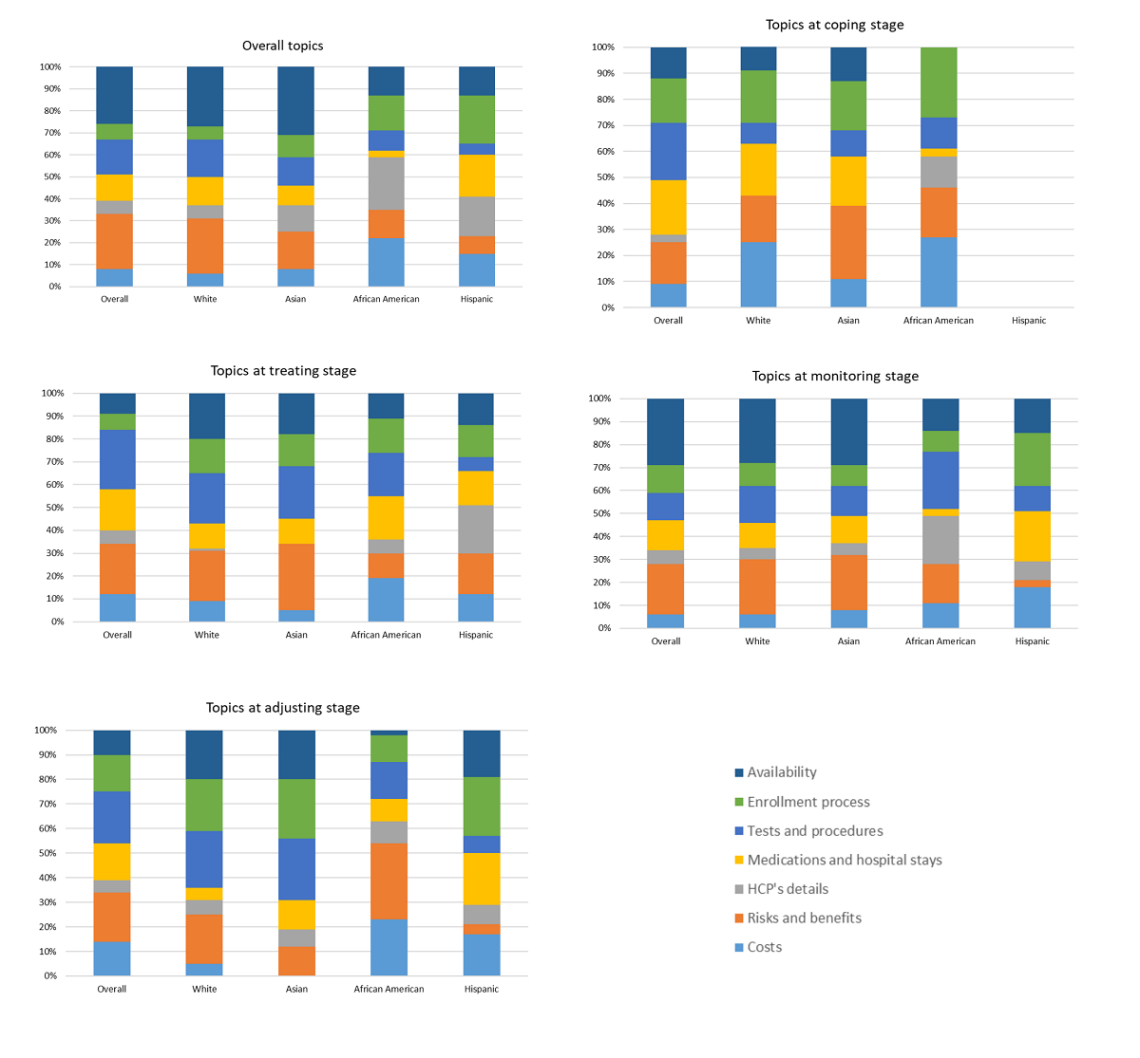
Comparison of top discussion topics by treatment stage and race or ethnicity. HCP: health care professional.

Analysis of the topics discussed identified seven main categories: (1) availability, (2) enrollment process, (3) tests or procedures, (4) medications or hospital stays, (5) HCP details, (6) risks, and (7) benefits and costs. A detailed analysis of these topics across treatment stages and racial or ethnic groups is shown in Figure 5. There are some distinct differences in the topics discussed overall by the different groups. African Americans and Hispanics discuss HCPs and cost and enrollment three times more often than the overall population. Hispanics also discussed medications and hospital stays 60% more often than other groups. Asians discuss HCPs twice as often as individuals who were White and the overall population (Figure 5).

The focus on HCP and costs persisted throughout the treatment process. At the coping stage, African Americans were 3 times more likely to discuss costs and four times more likely to discuss HCP details. As Hispanics did not often share at this stage in our analysis, this method cannot evaluate their concerns at this stage. Asians, however, were less likely to discuss tests and procedures and 1.7 times more likely to discuss risks and benefits.

At the treatment stage, all groups were more likely to discuss the enrollment process. African Americans were 3.5 times more likely to discuss HCP details, and White individuals and Asians were twice as likely to discuss clinical trial availability.

At the monitoring stage, cost is more likely to be discussed by African Americans and Hispanics (2 and 3 times more, respectively). HCP details (3.5 times more) and tests or procedures (2 times more) were also discussed more by African Americans, whereas medications, hospital stays, and enrollment processes were discussed more by Hispanics.

At the adjusting stage, White individuals, Asians, and Hispanics discussed clinical trial availability and enrollment more often than African Americans. Modest differences were also seen in discussions about cost (1.6 times more), risk benefits (1.5 times more), and HCP details (two times more) for African Americans.

### Barriers to Clinical Trial Enrollment

**Figure 6:**
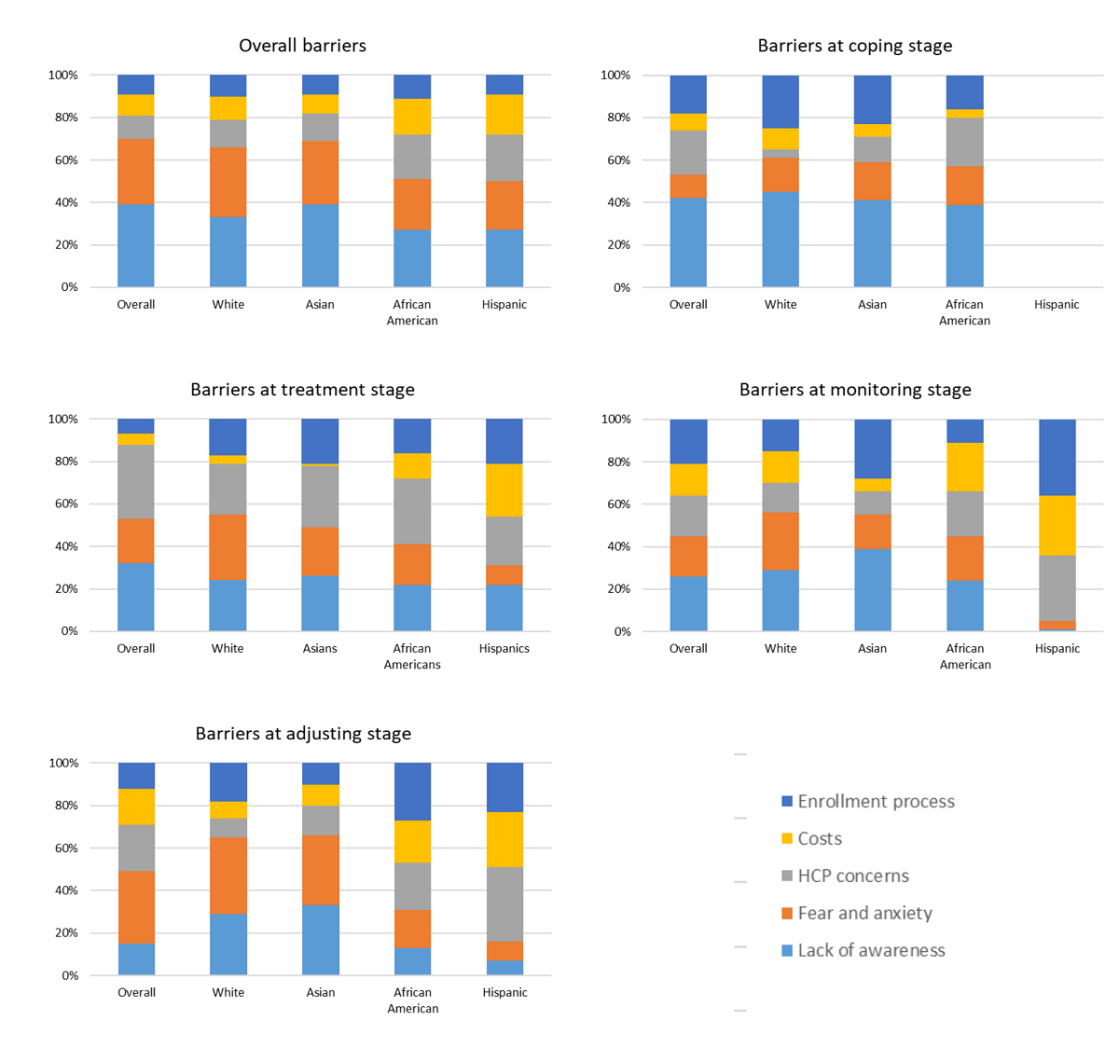
Comparison of clinical trial barriers by treatment stage and race or ethnicity. HCP: health care professional.

Barriers identified from the discussions included (1) lack of awareness, (2) fear and anxiety, (3) HCP concerns, (4) costs, and (5) the enrollment process. Overall, lack of awareness and fear and anxiety are the biggest barriers discussed by White individuals and Asians, whereas African Americans and Hispanics were more likely to discuss HCP concerns (three times more). The enrollment process is also more of a concern for African Americans and Hispanics (2 times and 3 times more than overall, respectively), and cost was discussed twice as often among Hispanics (Figure 6).

When analyzed by treatment stage, White individuals discussed HCP less frequently at the coping stage (5.2 times less) than Asians or African Americans. As noted above, Hispanics did not share at this stage, so their discussions were not available for analysis. At the treatment stage, costs became a major barrier discussed by Hispanics (discussed 5 times more) and, to a lesser extent, African Americans (2.6 times). The enrollment process became more of a concern for all groups. At the monitoring stage, Hispanics discussed the lack of awareness less frequently and increased discussions on cost, HCP, and enrollment issues. Posts by African Americans were more likely to mention cost as a barrier, whereas Asians discussed awareness and White individuals discussed fear. At the adjusting stage, significant differences were seen in the ranking of barriers, with White individuals and Asians noting more fear and less awareness, whereas African Americans were more likely to discuss HCPs, costs, and enrollment barriers.

## Discussion

### Principal Findings

In this study, we used a powerful new digital search technology to examine web-based social media posts for sentiments, top topics of interest, and barriers to enrollment in cancer clinical trials across varying racial and ethnic populations.

This type of social media analysis is innovative in its ability to mine large amounts of unstructured data and in its use of pattern recognition and adaptability instead of structured model assumptions. The ability to mine hundreds of thousands of web-based discussions across tens of thousands of sites allows us to nonintrusively capture spontaneous, real-time conversations about clinical trials from diverse populations. The high volume of discussions analyzed in this study gives us a unique opportunity to begin to thoroughly dissect these concerns by treatment stage and race or ethnicity and better understand the sentiments and feelings of the groups throughout the cancer care process.

In our analysis, issues related to HCP interactions, cost of care, fear, anxiety, and lack of awareness dominate the discussions among all racial and ethnic groups, but there are notable differences in the frequency of these topics and barriers discussed on the web by different populations. There is a wealth of literature on the barriers minorities face in clinical trial enrollment, and our data reiterate many of those findings, especially the significant roles of the HCP and patient relationship and discrepancies in the financial burden of care [18-21].

HCPs are the first line of interaction with cancer patients and are meant to fill in information gaps that patients have about their complete care, including disease expectations, treatment, symptom management, and costs of care. Unfortunately, data from this study and others show that some patients are not satisfied as they are not able to meet their needs. Although this study did not identify specific HCP issues, other studies have reported specific concerns. For example, many community physicians that minorities are most likely to access are not equipped to provide adequate information on these topics. These HCPs do not have the knowledge and/or time to research clinical trial options for all patients and lack adequate administrative support to assist patients with the enrollment and eligibility requirements [18,20]. Furthermore, many studies have noted implicit bias of physicians toward minorities, which may hinder appropriate discussions of clinical trial risks and benefits. These studies found that physicians may presume that minority patients cannot adhere to trial regulations or fear patient rejection from the trial [18,21]. Importantly, many clinical trials are not adequately designed to account for differences in baseline organ function and comorbidities that can differ between minority groups, leading to the failure of these patients to meet enrollment criteria [22,23].

Another significant barrier in the patient and provider relationship is trust. Minorities, especially African Americans, are much more likely to mention provider distrust as a health care barrier. This phenomenon has roots in the inequities in health care that these groups have experienced and past unethical research practices by the research community [24]. A recent meta-analysis by Hurd et al [25] examined the role of patient trust in oncology clinical trials and found that distrust of HCPs is most prominent at the periods of care transition, that is, transferring from a community physician to a cancer clinic for treatment and back again for monitoring or surveillance by the community physician. This is reflected in our data, particularly for African Americans, as illustrated in Figures 5 and 6, with increases in HCP concerns peaking at the coping and monitoring stages.

With increased recognition of the lack of diversity in clinical trials, considerable progress has been made in finding ways to better connect minority populations with clinical trial opportunities. The FDA recently published guidance on enrollment practices, eligibility criteria, and clinical trial design to enhance diversity [26]. Outreach by clinical trial sites to community physicians, leadership roles, committees committed to diversity, cultural training of physicians, community advisory boards and lay community representatives, culturally literate patient navigators, and culturally appropriate patient education [27-30] are all steps that have been shown to have a positive effect on minority clinical trial enrollment [18-21]. Recommendations to address minority distrust of HCPs include provider and support staff diversity, discussion of research transparency, and statement of overall clinical trial goals. The community mindset of some populations can also be leveraged to emphasize altruism and benefits to the community [31].

Besides HCPs and enrollment concerns, we found treatment cost to be a disproportionate concern for African Americans and Hispanics. This correlates with current research on minority clinical trial barriers [32-34] and is a significant topic that needs to be addressed. Direct costs of treatment are often covered by insurance policies under the requirements of the Affordable Care Act; however, older grandfathered plans and Medicaid often do not cover National Cancer Institute–designated centers in the network [33,34]. In addition to treatment costs, indirect care costs such as travel and lodging for patients who reside far from the treatment center are also of great concern. Some studies have shown that financial assistance plans increased enrollment of low-income and rural patients with financial barriers related to lodging and travel and that this intervention decreased this specific patient concern throughout their treatment process [35]. The widespread use of financial assistance has been limited, however, because of ethical concerns regarding the coercion of financially burdened patients to participate. The American Society of Clinical Oncology has issued recommendations on clinical trials to include health policy changes, cost transparency, clear incentives that do not coerce, and improved cost data [32].

### Limitations

There are many limitations to this type of study. Although we were able to identify demographic information for many of the posters, more than half of the posters were unidentifiable. There may be a bias to the posts that were identifiable, and misidentification events are possible. Second, the feelings expressed in web-based forums may be different or skewed more negatively than feelings expressed elsewhere, such as during physician visits. In addition, whereas we did not include multiple posts by a single user within a thread or conversation, if users posted in multiple threads or on multiple sites, they may have been counted multiple times. We did not have access to other avenues that patients use offline to discuss and share information, which is evident in the lack of data on Hispanics in the coping stage, which may not allow us to obtain a complete analysis of all groups at all stages of treatment. Finally, we did not have information on clinical diagnoses, treatment offerings, treatment adherence, or outcomes for patients that would affect their sentiments.

### Conclusions

Overall, this study provides detailed insights into the content and sentiments of web-based discussions regarding clinical trials. This information is valuable for identifying the ideal content and timing for the delivery of clinical trial information and resources for different racial and ethnic groups. Information on feelings and sentiments reveals opportunities to leverage hopeful and empowered feelings and dispel fears and misconceptions about clinical trial participation. Detailed information on clinical trial barriers, including distrust of HCPs, financial disparities, and the need for tailored education and enrollment assistance for minorities, is useful for developing strategies, policies, and practices to minimize health care inequality and increase the recruitment of minorities into clinical trials.

## Abbreviations

HCP: health care professional
